# Gas Recognition in E-Nose Systems: From Sensing Strategies to Reliable Pattern Recognition

**DOI:** 10.3390/s26206359

**Published:** 2026-10-09

**Authors:** Antonio Pardo, Luis Fernández

**Affiliations:** 1 Department of Electronics and Biomedical Engineering, Universitat de Barcelona, 08028 Barcelona, Spain; lfernandez@ub.edu; 2 Institute for Bioengineering of Catalonia (IBEC), The Barcelona Institute of Science and Technology, 08028 Barcelona, Spain

## 1. Introduction

Electronic noses (e-noses) are analytical systems designed to recognize complex volatile patterns through the combined use of sensing elements and data-processing methods. Their development has traditionally relied on arrays of partially selective chemical sensors together with multivariate analysis or pattern recognition techniques. Over time, the concept has expanded to include alternative sensor-operating modes, separation stages, and instrument-based approaches that generate richer and more complex datasets.

This evolution has also changed the way gas recognition is approached. Performance no longer depends only on the sensing material or on the choice of classifier. It is determined by several connected stages, including sample handling, measurement protocol, sensor operation, signal preprocessing, feature extraction, statistical or machine learning modelling, and validation. The contributions collected in this Special Issue reflect this broader view of the e-nose system.

The Special Issue includes six original research articles and one review. Four contributions address food quality, food safety, or broader agri-food applications. Borowik et al. investigated the discrimination of wheat grains infected by different Fusarium species; Skowronkova et al. developed a low-cost system for the separation and detection of volatile compounds relevant to food analysis; Fernandez et al. focused on olive oil quality classification using an instrument-based e-nose; and Martínez et al. explored the early detection of fungal spoilage in plums.

Two contributions concern biomedical applications. Sansone and Tonacci provide a review of e-nose approaches for kidney disease assessment, whereas Dragonieri et al. investigate the reproducibility and standardization of breathprints in healthy subjects. Finally, Szyłak-Szydłowski et al. address environmental monitoring through the assessment of odour emissions during composting under operational conditions.

Although the application domains differ, these studies share a common objective: extracting useful and reproducible information from complex volatile mixtures and converting it into an analytical decision.

## 2. Sensing and Measurement Strategies

A basic limitation of many e-nose systems is the partial selectivity of the sensing elements. Different strategies have been developed over the years to increase the amount of useful information obtained from such sensors. The contributions to this Special Issue illustrate several of these approaches, including active control of sensor operating conditions, pre-separation before detection, and the use of instrument-based systems.

Temperature modulation is one established strategy for increasing the information obtained from metal-oxide gas sensors. Borowik et al. applied this approach to commercially available sensors in a low-cost e-nose designed to discriminate wheat grains infected by four Fusarium species. Features extracted during temperature modulation provided better classification performance than those obtained exclusively from the conventional adsorption phase, while combining features from both response modes produced a further improvement. The resulting Support Vector Machine model achieved an overall four-class classification rate of 70%, with Fusarium poae being more readily distinguished from the other species.

The interest of this work therefore lies not in temperature modulation itself, which is a well-established approach in gas sensing, but in its implementation in a low-cost e-nose and in the use of information from different phases of the sensor response. It illustrates how the measurement protocol can influence the discriminative information available to the recognition model even when the sensor hardware remains unchanged.

A different strategy was explored by Skowronkova et al., who introduced a separation stage before detection. Their system combined a 3D-printed capillary column with a MiCS-6814 chemiresistive sensor. The optimized configuration distributed the sensor response in time and allowed peaks associated with ethanol and methanol to be distinguished. The study also identifies several steps needed for further development, including quantitative analysis, measurements of more complex mixtures, miniaturization, and the integration of the components into a more compact system.

Fernandez et al. address the same general problem from another perspective by using multi-capillary column ion mobility spectrometry (MCC-IMS). In this case, chromatographic separation and ion mobility detection generate multidimensional volatile fingerprints with greater chemical resolution than that typically provided by conventional gas-sensor arrays. However, the richer measurement also increases the importance of data preprocessing and modelling.

These studies represent different ways of increasing the information available for recognition: exploiting dynamic sensor responses, separating mixture components before detection, or using analytical instrumentation that generates multidimensional signals. In all cases, the usefulness of the additional information depends on how it is subsequently processed.

## 3. Signal Processing and Pattern Recognition

Signal processing is therefore a central part of the e-nose workflow. The raw output of a sensor array or an instrument-based system is rarely the final representation used for recognition. Preprocessing, feature extraction, dimensionality reduction, and statistical modelling determine which components of the signal are retained and how they are used.

Fernandez et al. provide a direct example of this issue. Their work proposes an untargeted multivariate workflow for MCC-IMS data that avoids dependence on conventional peak integration procedures. The preprocessing strategy reduced the number of latent variables required to obtain stable PLS-DA performance from ten to five. Baseline correction had the largest effect on the final model, while signal alignment also contributed. The resulting models were evaluated on previously unseen olive oil samples and achieved an accuracy of 83% ± 5%.

The work of Borowik et al. also shows how the representation of the sensor response affects classification. Different results were obtained depending on whether the predictors were derived from the adsorption phase, the temperature modulation phase, or a combination of both. This reinforces the idea that the performance of an e-nose cannot be attributed only to the sensor array or to the final classifier. Feature generation is part of the recognition process.

The contributions also illustrate different roles for multivariate analysis. Martínez et al. used Linear Discriminant Analysis to distinguish healthy plums from those inoculated with Monilinia laxa. Under the experimental conditions considered, the LDA models achieved complete discrimination between healthy and infected samples, including at an early stage of contamination.

Dragonieri et al., in contrast, used Principal Component Analysis and clustering to study the internal consistency of breathprints in healthy subjects rather than to build a disease classifier. Four principal components explained 97.15% of the variance, and 138 of the 139 subjects formed a closely grouped cluster. In this case, multivariate analysis was used to examine reproducibility and to establish whether a stable reference pattern could be obtained under standardized conditions.

These examples show that pattern recognition methods in e-nose research can serve different purposes, including classification, exploratory analysis, dimensionality reduction, and assessment of reproducibility. The choice of method should therefore follow the analytical question rather than being treated as an independent optimization problem.

## 4. Validation, Reproducibility, and Standardization

The studies in this Special Issue also underline the difference between obtaining good classification results within a particular dataset and demonstrating that a model will generalize to new measurements.

This distinction is especially important in e-nose research because datasets are often relatively small while the number of measured or derived variables can be large. Under these conditions, model selection and preprocessing can lead to optimistic performance estimates if the validation procedure is not properly separated from model development.

Fernandez et al. explicitly address this issue by incorporating a validation scheme intended to limit overfitting, optimize model parameters, and estimate performance on unseen samples. Their results also show that preprocessing can reduce model complexity while retaining predictive performance.

In biomedical applications, the problem extends beyond statistical validation to measurement standardization. Sansone and Tonacci identify validation and standardization as unresolved limitations for the clinical use of e-nose systems. Their review considers the need to validate not only the prediction model but also the instrument and the complete analysis pipeline, including potential sensor failures and recalibration requirements. The review also points to the need for larger datasets, improved sensing technologies, and well-defined validation procedures if e-nose methods are to be used more broadly in kidney disease assessment.

Dragonieri et al. examine another aspect of standardization by studying the reproducibility of breathprints in a healthy population. Their results show a high degree of similarity among the measurements obtained from 139 subjects under a standardized protocol. The authors nevertheless note that broader validation should include more diverse populations, pathological cohorts, multiple devices, and studies of long-term stability.

Taken together, these studies suggest that validation should be considered part of the design of an e-nose study rather than a final statistical step. Independent test samples, external validation when feasible, repeated measurements over time, inter-device comparisons, standardized sampling procedures, and clear reporting of preprocessing and model selection procedures are all relevant to the assessment of system performance.

## 5. Complementarity with Reference Analytical Methods and Real-World Deployment

Several contributions also examine the relationship between electronic noses and established reference methods. E-noses offer advantages in terms of speed, operational simplicity, portability, and the possibility of repeated or near-real-time measurements. However, their pattern-based operation often benefits from comparison with analytical methods that provide chemical identification or a recognized reference measurement.

Martínez et al. combined e-nose measurements with GC/MS characterization of the volatile profiles associated with Monilinia laxa infection in plums. The study found a relationship between the sensor responses and the changes in volatile composition associated with fungal contamination. The authors also identify external validation with naturally infected commercial fruit, measurements under refrigerated conditions, and shorter detection times as necessary steps before broader application.

In biomedical applications, Dragonieri et al. similarly note that GC-MS would be useful for compound-level validation of the breathprints detected by the e-nose. Their study uses the global VOC pattern rather than identifying the individual compounds responsible for that pattern, and future work combining both approaches could help establish the chemical basis of the measured signatures.

Szyłak-Szydłowski et al. provide an example of measurements performed under field conditions. Their study compared three approaches during composting: field olfactometry, VOC measurements, and a PEN3 e-nose equipped with ten metal-oxide sensors. The composting process showed repeatable temporal patterns, and several e-nose sensor responses correlated with the logarithm of VOC concentration, with coefficients above |r| = 0.7. The measurements also allowed three phases of the process to be distinguished.

The authors also state that the amount of data collected was not sufficient to justify more complex machine learning modelling at this stage. This distinction is important: demonstrating correlations under operational conditions is a different objective from developing and validating a predictive model.

These studies suggest that e-noses and reference analytical methods can play complementary roles. Reference techniques can support chemical interpretation, calibration, and validation during development, whereas e-noses can provide faster and simpler measurements once the analytical relationship has been established.

## 6. Future Directions

Several research directions emerge from the contributions to this Special Issue.

One concerns the joint design of sensing and data processing. Dynamic sensor operation, pre-separation, and multidimensional analytical measurements can all increase the amount of information available, but the benefit depends on the preprocessing and modelling strategy used to extract that information.

A second issue is generalization. Models developed using a particular device, set of samples, or measurement protocol need to be evaluated under new conditions. This includes external sample sets, different instruments, long-term measurements, and realistic operating environments. The biomedical contributions place particular emphasis on standardization, while the agri-food and environmental studies point to the importance of moving from controlled experiments toward naturally occurring samples and field measurements.

Model complexity is another consideration. More complex machine learning and deep learning approaches may be useful when the available datasets support them, but complexity should not substitute for validation. In many applications, a simpler model may be preferable if it provides comparable performance and can be more easily interpreted, validated, and transferred between measurement systems.

Finally, practical deployment will depend on factors such as miniaturization, automation, sensor stability, device-to-device reproducibility, cost, and maintenance. These issues appear explicitly in the low-cost systems described by Borowik et al. and Skowronkova et al., but they are also relevant to clinical and environmental applications.

## 7. Conclusions

The contributions gathered in this Special Issue show that gas recognition in e-nose systems depends on several connected stages, from sensing and sample handling to signal processing, pattern recognition, and validation.

The studies included here show that improvements can arise at different points in this chain. Temperature modulation can provide additional information from conventional gas sensors; pre-separation can help distinguish components before detection; instrument-based approaches can provide multidimensional fingerprints; and preprocessing can reduce model complexity while retaining predictive performance.

The applications represented in the Special Issue also show that the main challenges are not limited to classification accuracy. Reproducibility, standardization, external validation, long-term stability, and performance under realistic conditions remain important for translating laboratory studies into routine use.

A common conclusion from these contributions is that gas recognition should be treated as a system-level problem. Performance depends on the combined design of the sensing strategy, measurement protocol, data-processing workflow, recognition model, and validation procedure.

We hope that the studies collected in this Special Issue will contribute to the development of e-nose systems that provide reproducible and transferable results across food analysis, biomedical applications, environmental monitoring, and related fields.

We would like to thank all the authors for their contributions and the reviewers for their careful evaluations and constructive comments.

